# Drug-resistant *Neisseria gonorrhoeae* in Michigan

**DOI:** 10.3201/eid1107.041359

**Published:** 2005-07

**Authors:** Kathryn E. Macomber, Martha S. Boehme, James T. Rudrik, Dara Ganoczy, Erin Crandell-Alden, William A. Schneider, Patricia A. Somsel

**Affiliations:** * Michigan Department of Community Health, Lansing, Michigan, USA;; † Department of Veterans Affairs, Ann Arbor, Michigan, USA

**Keywords:** Neisseria gonorrhoeae, Drug Resistance, microbial, Epidemiology, Quinolones, Therapeutics, Risk Factors, Pilot Projects

## Abstract

The increasing prevalence of quinolone-resistant *Neisseria gonorrhoeae* (QRNG) in the United States is a cause for concern. Detecting resistance is complicated by the widespread use of molecular tests that do not provide isolates for susceptibility testing. The Michigan Department of Community Health developed a sentinel surveillance program to detect antimicrobial drug resistance in *N. gonorrhoeae*. Sentinel surveillance from 11 laboratories submitted 1,122 isolates for antimicrobial drug susceptibility testing and detected 2 clusters of QRNG from January 2003 to September 2004. These clusters were epidemiologically distinct: one involved young, heterosexual youth, and the other involved older men who have sex with men. This finding led to changes in local treatment recommendations that limited spread of resistant strains. Development of the sentinel program, collection of data, and epidemiologic analysis of the clusters are discussed.

Since the 1970s, the treatment and control of gonorrhea have been complicated by the ability of *Neisseria gonorrhoeae* to develop resistance to a variety of antimicrobial agents, including penicillin, tetracycline, and fluoroquinolones. Recent limitations in oral treatments for gonorrhea resulting from the discontinued manufacturing of cefixime, as well as decreases in the availability of isolates for susceptibility testing as culture methods are replaced by nucleic acid amplification tests, pose additional challenges for successfully treating patients and identifying resistant organisms.

In 1986, the then-Centers for Disease Control (CDC) established the Gonococcal Isolate Surveillance Project (GISP) to monitor changes in antimicrobial drug–susceptibility patterns. Twenty-five isolates are collected monthly from participating sexually transmitted disease (STD) clinics across the United States (30 cities represented in 2003) and are sent to CDC for susceptibility testing (*1*).

In the early 1990s, fluoroquinolone-resistant *N. gonorrhoeae* (QRNG) was reported from a number of areas outside of the United States, and resistant strains became well established in Thailand, Hong Kong, Japan, and the Philippines. Sporadic reports of QRNG in the United States at that time were usually associated with travel to Asia. Prevalence of QRNG in Hawaii steadily increased from 1997 to 2001 (*2*,*3*). In 2000, California reported QRNG in San Francisco, San Diego, and Orange County. In 2001, 33 (2.5%) of 1,311 of isolates tested in California were resistant to fluoroquinolones; this increase continued in 2002 (*4*,*5*). As a result of the increasing prevalence of QRNG, the Hawaii Department of Health and California Department of Health Services recommended that clinicians avoid using fluoroquinolones when treating gonorrhea (*6*). Because of QRNG prevalence variation in countries outside of the United States, CDC recommended in its 2002 STD Treatment Guidelines that fluoroquinolones not be used to treat gonorrhea acquired in Asia, the Pacific Islands, Hawaii, California, or other areas with an increased prevalence of QRNG (*6*–*9*).

In addition to Hawaii and California, low numbers of QRNG-resistant isolates had been reported from cities in the United States before 2000 (*4*,*5*). GISP data for 2003 showed resistant isolates from Cleveland, Baltimore, Chicago, Dallas, and Kansas, with a significant increase in QRNG in Seattle, New York City, Massachusetts, California, and Michigan, and smaller increases in Phoenix, Minneapolis, Chicago, Las Vegas, and Portland (*1*).

The primary therapies currently recommended by CDC for uncomplicated gonococcal infections of the cervix, urethra, and rectum include ceftriaxone, cefixime, or a fluoroquinolone (ciprofloxacin, levofloxacin, or ofloxacin) (*6*). In July of 2002, Wyeth Pharmaceuticals stopped manufacturing cefixime, the only recommended oral cephalosporin; company inventories were fully depleted by October 2002 (*10*). Michigan used only cefixime tablets, although other states may have used cefixime oral suspension, which may have been available longer. Although the US Food and Drug Administration has approved cefpodoxime and cefuroxime axetil to treat uncomplicated gonococcal infections, CDC has not recommended either of these oral cephalosporins to replace cefixime because they fail to meet CDC's efficacy standards (*10*).

CDC recommended, in 2002, that state health departments monitor local antimicrobial drug–susceptibility patterns to guide local treatment recommendations (*3*). In response, the Michigan Department of Community Health (MDCH) established a sentinel surveillance system to monitor the prevalence of drug-resistant gonorrhea, characterize patients with drug-resistant infections, and provide local treatment recommendations in Michigan. Before 2003, only sporadic cases of QRNG were detected in Michigan; all of these patients acquired their infections during foreign travel. However, resistant strains might have gone undetected, as an estimated 97% of genital gonorrhea testing in Michigan is performed by nucleic acid testing, from which viable isolates for susceptibility testing cannot be obtained (MDCH, unpub. data). To augment routine susceptibility studies performed at MDCH, a special surveillance project to collect gonococcal isolates from clinical laboratories across the state was initiated in July 2002 and continued through September 2004. We describe the development of this surveillance project, discuss the challenges of maintaining surveillance on a voluntary basis, and present data collected from the project.

## Methods and Materials

### Selection of Participating Sites

Although ≈110 clinical laboratories in Michigan offer comprehensive microbiology services, many have switched to nucleic acid methods. To determine the number of laboratories that perform genital gonorrhea cultures as their routine detection method, positive gonorrhea case reports submitted to the state health department during a representative 3-month period (August–October 2001) were examined to obtain a convenience sample of laboratories that culture genital specimens for gonorrhea. Nineteen laboratories reported ≥1 culture-based positive results during that time. These laboratories were contacted to determine the average number of cultures positive for gonorrhea per year, whether cultures were performed on genital specimens, whether genital gonorrhea cultures were expected to continue to be collected for the next 6 months, and whether the laboratory was willing to submit isolates for surveillance. Of the 19 laboratories, 5 clinical (hospital) laboratories were identified that recovered ≥10 genital gonorrhea cultures each month; all 5 agreed to participate as sentinel sites. These 5 laboratories were located in 5 different counties (Table 1). The state health department laboratory also received routine gonorrhea cultures from a local health department STD clinic and occasionally referred *N. gonorrhoeae* isolates for identification or susceptibility testing. These cultures were also included in the surveillance system. After the identification of QRNG cases, surveillance was expanded to include 4 additional STD clinics in 4 counties (Table 1).

**Table 1:** Characteristics of submitting providers, gonorrhea testing, Michigan

Laboratory	Location	Types of sites*	Location	2003 participants N = 564 (%)	2004 participants N = 510 (%)
967-bed teaching hospital	Southeast	ER, PMD	Southeast	45 (8)	32 (6)
181-bed community hospital	West	ER, PMD	West	104 (18)	N/A
438-bed teaching hospital	Mid-state	ER, PMD	Mid-state	57 (10)	N/A
County A	Mid-state	STD and teen clinic	Mid-state	136 (24)	104 (20)
County B	West	STD clinic	West	22 (4)	112 (22)
360-bed community hospital	Southwest	ER, PMD	Southwest	84 (15)	N/A
County C	Southeast	STD clinic	Southeast	54 (10)	174 (34)
County D	East	STD clinic	East	17 (3)	33 (6)
County E	East	STD clinic	East	N/A	23 (5)
478-bed community hospital	Southeast	ER, PMD	Southeast	13 (2)	9 (2)
University student health center	Southeast	Student health	Southeast	15 (3)	9 (2)
Referred from other sites	Varied	ER, PMD, STD clinic,	Varied	17 (3)	14 (3)

*ER, emergency room; PMD, primary medical doctor; STD, sexually transmitted disease; N/A, not available.

### Isolation, Identification, and Susceptibility Testing

Laboratories were provided with chocolate agar slants (Remel, Lenexa, KS, USA), International Air Transport Association–compliant mailing containers, and specific instructions for packaging and shipping. Courier pickup of isolates on an on-call basis was also arranged. Isolates were accepted on chocolate agar slants or frozen in Trypticase soy broth with glycerol.

Gonococcal isolates from genital and nongenital cultures either were recovered from cultures collected by the local health department STD clinic or were referred from the sentinel sites or other clinical laboratories. Cultures obtained from the local STD clinic were plated onto Modified Thayer-Martin medium (Becton Dickinson, Cockeysville, MD, USA) and incubated for 18 to 24 h at 35°C in a candle jar before transport to MDCH. At MDCH, the plates were incubated an additional 48 h at 35°C in 5% to 10% CO_2_ and examined daily. Suspect colonies grown on Thayer-Martin medium and referred isolates were presumptively identified by Gram stain and oxidase reaction. The isolates were subcultured on chocolate agar (Remel) for further testing. All isolates were frozen in skim milk at –70°C.

Isolates were identified by using the apiNH system (bioMérieux, Inc., Durham, NC, USA). If an isolate was not identified by apiNH, conventional biochemical tests were performed, including cystine tryptic agar sugar fermentation test with glucose, sucrose, maltose, and lactose.

Antimicrobial drug susceptibility for ciprofloxacin, spectinomycin, tetracycline, ceftriaxone, and cefixime or cefpodoxime was determined by disk diffusion on gonococcal (GC) agar base supplemented with 1% GCHI enrichment (Remel) according to the Clinical and Laboratory Standards Institute (formerly NCCLS) procedure (*11*). Any isolate categorized as repeatedly resistant to or intermediately resistant to ciprofloxacin was tested to determine MIC (*12*). MIC was determined by Etest (AB BIODISK North America, Piscataway, NJ, USA) on GC Agar Base supplemented with 1% GCHI enrichment, according to the manufacturer's instructions (*13*). *N. gonorrhoeae* ATCC 492226 was used as the quality control strain for both disk diffusion and Etest. Beginning January 1, 2004, MDCH added cefpodoxime and deleted cefixime from its routine susceptibility-testing panel for gonorrhea.

### Epidemiologic Analysis

Final reports of susceptibility results were distributed to the submitting laboratory and to the MDCH Bureau of Epidemiology. For cases with susceptibility testing results reported from January 2003 to September 2004, provider information was obtained from the submitting laboratory. Demographic, behavioral, and clinical data were solicited from the patients' healthcare providers. For all gonorrhea patients, each provider was contacted to give permission to receive a data collection form by secure fax. The form collected information on reason for visit, zip code, age, race, ethnicity, sex, sexual orientation, prior gonorrhea infection, primary therapies for gonorrhea and chlamydia, and the reason a culture was performed. The completed forms were faxed back to the Bureau of Epidemiology, where epidemiologic and laboratory data were entered into a Microsoft Access database (Microsoft Corp., Redmond, WA, USA). Patients with QRNG were interviewed (by phone or in person) by MDCH disease intervention specialists, and additional information was collected, including that on illicit drug use, recent use of antimicrobial agents, sexual partner risk factors, HIV status, and travel history. Prevalence ratios were used to examine the associations between QRNG and demographic, behavioral, and clinical characteristics. Data were analyzed with SAS version 9.1 (SAS Institute, Inc., Cary, NC, USA). The project was determined to be routine surveillance activity and thus exempt from human subjects review by the MDCH Institutional Review Board.

## Results

From January 1, 2003, to September 30, 2004, susceptibility testing results for 1,122 isolates (from 1,074 patients) were reported. Patient and specimen characteristics for QRNG and non-QRNG isolates are shown in Table 2, stratified by year. A total of 582 isolates (from 564 patients) obtained by disk diffusion were reported during calendar year 2003. All isolates were susceptible to cefixime, ceftriaxone, and spectinomycin; 43 (7.4%) were resistant to tetracycline. Seventeen (2.9%) isolates were resistant to ciprofloxacin (MIC ≥1 μg/mL), and 1 (0.2%) showed intermediate resistance (MIC 0.12–0.5 μg/mL). The 17 ciprofloxacin-resistant isolates in 2003 represented 14 individual cases of QRNG. Table 3 lists 2003 and 2004 QRNG cases with relevant risk factors and demographics. In 2003, 11 patients were male. Patients ranged in age from 16 to 45 years (median 26). Nearly half of the QRNG patients were white (43%). Four of the 11 male patients were men who have sex with men (MSM). A large number of cases (57%) were detected at public STD clinics in county A.

**Table 2:** Patient and specimen characteristics, gonorrhea study, Michigan*

	2003 non-QRNG N = 550 (%)	2003 QRNG N = 14 (%)	2004 non-QRNG N = 502 (%)	2004 QRNG N = 8 (%)
Median age, y	23	26	24	26
Sex				
Male	267 (49)	11 (79)	411 (82)	8 (100)
Female	281 (51)	3 (21)	91 (18)	0
Unknown	2 (0)	0	0	0
Race				
Black	323 (59)	7 (50)	313 (62)	0
White	76 (14)	6 (43)	89 (18)	7 (88)
Other	9 (1)	0	6 (1)	1 (12)
Unknown	142 (26)	0	94 (19)	0
Asian	0	1 (7)	0	0
Hispanic				
Yes	11 (2)	1 (7)	16 (3)	0
No	204 (37)	12 (86)	283 (57)	7 (88)
Unknown	335 (61)	1 (7)	203 (40)	1 (12)
Site of specimen				
Cervix/vagina	260 (47)	2 (21)	74 (15)	0
Eye	1 (1)	1 (7)	1 (0)	0
Urethra	244 (44)	7 (43)	392 (78)	6 (75)
Throat	16 (3)	1 (7)	16 (3)	0
Rectal	17 (3)	1 (7)	11 (3)	1 (12)
Urine	6 (1)	2 (15)	5 (1)	1 (12)
Other	6 (1)	0	3 (0)	0
Symptoms				
Discharge/dysuria	338 (61)	10 (72)	373 (74)	6 (75)
None	72 (13)	3 (21)	56 (11)	1 (12)
Unknown	140 (26)	1 (7)	73 (15)	1 (12)

*QRNG, quinolone-resistant *gonorrhoeae*. †Intermediate QRNG grouped with non-QRNG cases.

**Table 3:** Quinolone-resistant gonorrhea cases, Michigan, 2003–2004

Collection date	Sex	Race	Age	Previous gonorrhea*	Sexual orientation	Site	Provider†
1/6/03	M	Black	16	No	Heterosexual	Urethra	Clinic A
3/5/03	M	Black	28	Yes	Heterosexual	Urethra	Clinic A
3/10/03	M	White	24	No	Heterosexual	Urethra	Clinic A
3/20/03	F	White	18	Yes	Bisexual	Cervix	Clinic A
5/19/03	M	Black	30	Yes	Heterosexual	Urethra	Clinic A
5/21/03	M	White	42	No	Heterosexual	Eye	PMD
7/5/03	M	Asian	31	No	Heterosexual	Urethra	PMD
7/8/03	M	White	19	No	Homosexual	Rectum	Clinic B
7/24/03	M	White	33	No	Homosexual	Throat	Clinic B
8/25/03	M	Black	36	Yes	Homosexual	Urethra	Clinic A
8/29/03	F	Black	18	No	Heterosexual	Cervix	Clinic A
8/26/03	M	Black	19	No	Heterosexual	Urine	Clinic A
9/26/03	F	Black	45	No	Heterosexual	Urethra	PMD
10/27/03	M	White	24	Unknown	Homosexual	Urine	PMD
1/22/04	M	White	43	Yes	Homosexual	Urethra	Clinic B
2/23/04	M	Other	39	Yes	Bisexual	Urine	PMD
3/15/04	M	White	20	No	Homosexual	Urethra	Clinic B
4/7/04	M	White	24	No	Homosexual	Urethra	Clinic B
5/14/04	M	White	26	No	Homosexual	Urethra	Clinic
6/28/04	M	White	21	Yes	Homosexual	Urethra	Clinic B
7/9/04	M	White	28	No	Homosexual	Rectum	Clinic B
8/18/04	M	White	26	No	Homosexual	Urethra	Clinic C

*Has the patient had gonorrhea (ever)? †PMD, primary medical doctor; clinic, county sexually transmitted disease clinic.

From January 1 to September 30, 2004, a total of 540 (510 patients) isolates whose susceptibilities were measured by disk diffusion showed no resistance to cefpodoxime, ceftriaxone, and spectinomycin. Eight isolates (1.5%) were resistant to ciprofloxacin, and 1 (0.2%) isolate had intermediate resistance. Resistance to tetracycline was similar to that seen in the previous year. All 8 cases of QRNG detected in the 2004 study period were in MSM. Patients ranged from 20 to 43 years of age (median 26). Most were white (88%), and more than half (63%) were detected at clinic B, a public STD clinic in a county not contiguous to clinic A.

Cumulative distribution, for the entire study period, of the submissions by site are shown in Table 1. Fifteen percent (158/1,074) of all gonococcal isolates were submitted by emergency rooms, 19% (205/1,074) were from primary medical doctors, and 64% (692/1,074) were from STD clinics. Although 35% of isolates were submitted by private providers, only 23% of QRNG cases were identified through those venues. Most QRNG cases were identified through public STD clinics (77%). The overall return rate of the data collection form from healthcare providers was 84%. Providers varied significantly in their response to a request for patient information; STD clinics and private medical doctors had higher response rates (93% and 72%, respectively) than emergency rooms (55%).

Although most persons with gonorrhea in our sentinel surveillance system are African American (76% of those with known race), the prevalence of QRNG was higher among whites, 7% versus 1% among non-whites. The prevalence ratio was 1.07 (p<0.0001, 95% confidence interval 1.02–1.11) (Table 4). The prevalence of QRNG was higher among those ≥40 years of age, 4% versus 2% among those <40 years of age. MSM constituted 11% of all gonorrhea patients in the surveillance system, but they accounted for 63% of male QRNG patients; heterosexuals comprised 37% of the male QRNG patients. The prevalence of QRNG was highest for MSM (14%) and was relatively low for heterosexual men (1%) and women (1%). The presence of symptoms was not associated with quinolone-resistant infections, as the prevalence of QRNG was similar between both those with and without symptoms. Prior history of gonorrhea was not significantly associated with QRNG; the prevalence of QRNG among those with a history of gonorrhea was 2%, versus 3% among those without a history of gonorrhea. Table 5 shows enhanced variables, such as drug use, travel history, and HIV status, collected from interviews conducted with QRNG patients. Most QRNG patients reported no recent use of antimicrobial agents, no illicit drug use, and no recent travel. These findings also held true for sex partner characteristics, although sex partners had an increased percentage of illicit drug use and a higher percentage of unknown responses. Three of the 22 QRNG patients during the study period were HIV-positive.

**Table 4:** Prevalence of ciprofloxacin-resistant gonorrhea infection among surveillance population

	% patients with QRNG (n/total)	Prevalence ratio (95% confidence interval)	p value
Sex		1.02 (1.00–1.04)	0.03
Male	3 (19/697)		
Female	1 (3/375)		
Men who have sex with men		1.14 (1.05–1.24)	<0.0001
Yes	14 (12/87)		
No	1 (7/488)		
White		1.07 (1.02–1.11)	<0.0001
Yes	7 (13/178)		
No	1 (9/896)		
Discharge and/or dysuria		0.991 (0.960–1.02)	0.57
Yes	2 (16/724)		
No	3 (4/131)		
History of gonorrhea		0.991 (0.967–1.02)	0.52
Yes	2 (7/285)		
No	3 (14/425)		
>40 y of age		1.02 (0.975–1.06)	0.30
Yes	4 (3/84)		
No	2 (19/990)		

**Table 5:** Characteristics of quinolone-resistant Neisseria gonorrhoeae patients, Michigan

	2003 N = 14 (%)	2004 N = 8 (%)
Primary therapy for gonorrhea		
Ciprofloxacin	0	2 (25)
Cefixime	3 (21)	0
Ceftriaxone	9 (64)	5 (63)
Other	2 (14)	0
None	0	1 (12)
Primary therapy for chlamydia		
Azithromycin	10 (72)	7 (88)
Doxycycline	1 (7)	1 (12)
None	3 (21)	0
Recent use of antimicrobial agent*		
Yes	2 (14)	1 (12)
No	9 (64)	6 (76)
Unknown	3 (21)	1 (12)
Illicit drug use		
Yes	3 (21)	1 (12)
No	8 (57)	6 (76)
Unknown	3 (21)	1 (12)
Recent travel†		
Yes	2 (14)	0
No	12 (86)	7 (88)
Unknown	0	1 (12)
HIV status		
Positive	1 (7)	2 (25)
Negative	13 (93)	6 (75)
Sex partner illicit drug use		
Yes	3 (21)	2 (25)
No	5 (36)	3 (38)
Unknown	6 (43)	3 (37)
Sex partner history of travel†		
Yes	1 (7)	0 (0)
No	10 (72)	5 (63)
Unknown	3 (21)	3 (37)
Sex partner recent antimicrobial use*		
Yes	1 (7)	0 (0)
No	7 (50)	5 (63)
Unknown	6 (43)	3 (37)

*Has the patient/sex partner used antimicrobial agents in the last 60 days? †Has the sex partner traveled outside the United States (or to Hawaii) in the last 60 days?

## Discussion

Michigan has seen a higher prevalence of QRNG in recent years among heterosexuals, especially men in county A (3.4%), compared to other surveillance sites, such as New York and Massachusetts (1.6% and 1.8%, respectively) (*14*). Shortly after sentinel surveillance was instituted, a geographic cluster of QRNG cases was discovered among a group of heterosexual teenagers in clinic A. A sexual link from 1 QRNG patient to another was discovered in only half of the patients. How QRNG initially emerged in this population is still unclear, as none of these heterosexual patients had a travel history. In cooperation with MDCH, the local health departments in county A (where the patients resided) and in the 5 contiguous counties issued a ban on using ciprofloxacin to treat gonorrhea. Before this recommendation, 71% of clinic A patients were treated with ciprofloxacin. After the recommendation was implemented, only 7% received ciprofloxacin as their primary treatment, according to clinic records.

A quick response to this geographic cluster may have halted the spread of QRNG in the community. However, at clinic A the direct costs of treatment increased, since 250 mg of intramuscular ceftriaxone costs nearly 3 times more than a 500-mg dose of ciprofloxacin (US $12.85 vs. $4.14). Indirect costs associated with staffing and increased amounts of time spent per patient (since all were treated with ceftriaxone and all had isolates submitted for culture) also increased but were not calculated. After 18 months without any cases, despite continued surveillance, the quinolone use ban on all patients (excluding MSM, per the revised STD treatment guidelines) was lifted, and no additional cases have since been reported. In addition, a cluster of cases in MSM in county B led local officials to make several recommendations for MSM, which included increasing provider awareness about the importance of asking about patients' sexual orientation, avoiding quinolone use, and using culture to diagnose gonorrhea.

During this surveillance project, 2 clusters were identified in 2 counties. However, QRNG surveillance is limited and not optimally representative: during the study period, it only operated in 9 of Michigan's 83 counties. This surveillance system captured only 4.4% of the total gonorrhea cases in Michigan; however, the counties represented in this surveillance system, in addition to the surveillance in Detroit City for the GISP project, report 38% of Michigan's gonorrhea cases. Those patients in the surveillance system are more likely to be seen at a public STD clinic (67% of those in the surveillance system vs. 23% of those in statewide morbidity reports), be African American (76% of the surveillance system vs. 40% of statewide morbidity), and male (67% of those in surveillance system vs. 44% of statewide gonorrhea case-patients). The Detroit City Health Department joined the GISP project in 2003, and no isolates collected from that site have been resistant to fluoroquinolones. This finding suggests that surveillance isolates should be collected from multiple geographic sites, ideally with demographic diversity, to demonstrate emerging resistance.

The fastidious nature of *N. gonorrhoeae* presented some challenges for the submitting laboratories. Approximately 10% of the total isolates received were either nonviable on subculture or overgrown with other organisms and were reported as unsatisfactory. At the end of December 2003, three of the 5 clinical laboratories stopped sending isolates to MDCH, citing economic and staffing barriers. Since QRNG prevalence was highest among patients seeking care from public STD clinics, MDCH asked additional public clinics to collect cultures. At the end of the study period, cultures were submitted by 5 local health departments and 2 private healthcare providers (a student health center and a major urban hospital laboratory).

MDCH will continue to provide routine *N. gonorrhoeae* susceptibility testing to monitor the emergence of resistance when isolates are available. Clinical laboratories are also encouraged to submit positive cultures to MDCH for susceptibility testing. To improve surveillance efforts, MDCH has recommended that clinicians culture specimens from patients with persistent symptoms. Clinical laboratories are asked to assist by submitting isolates for susceptibility testing from patients who are repeatedly culture-positive. However, as nucleic acid amplification tests replace culture-based methods, molecular techniques to demonstrate resistance and identify clusters will need to be developed.

Although Michigan's data are not geographically representative, the state's sentinel surveillance system is strong because numerator and denominator data are collected, allowing for the calculation of true prevalence ratios. The continued emergence of QRNG among gonorrhea cases will be a major financial limitation to state STD programs. Quinolones are currently the only oral treatment for gonorrhea recommended by CDC, and intramuscular ceftriaxone costs nearly 3 times more than a dose of ciprofloxacin. Since approximately one third of Michigan's estimated 17,000 gonorrhea patients in 2004 were treated in public clinics, the cost of QRNG will substantially limit the services the Michigan STD program can provide to residents. This study illustrates that, although a local ban on ciprofloxacin use in response to a QRNG cluster demanded more intensive resources for 1 Michigan county, the response may have been more timely, effective, and less costly than a statewide reaction.
